# Revitalising cancer trials post-pandemic: time for reform

**DOI:** 10.1038/s41416-023-02224-y

**Published:** 2023-03-23

**Authors:** Cienne Morton, Richard Sullivan, Debashis Sarker, John Posner, James Spicer

**Affiliations:** 1 Department of Medical Oncology, Guy’s & St Thomas NHS Foundation Trust, London, UK; 2 Institute of Cancer Policy, King’s College London, London, UK; 3 School of Cancer & Pharmaceutical Sciences, King’s College London, London, UK

**Keywords:** Phase I trials, Phase II trials, Health care economics, Cancer therapy, Adaptive clinical trial

## Abstract

The COVID-19 pandemic posed significant risk to the health of cancer patients, compromised standard cancer care and interrupted clinical cancer trials, prompting dramatic streamlining of services. From this health crisis has emerged the opportunity to carry forward an unexpected legacy of positive reforms to clinical cancer research, where conventionally convoluted approvals processes, inefficient trial design, procedures and data gathering could benefit from the lessons in rationalisation learned during the pandemic.

## Introduction

Cancer is a leading contributor to health and economic burdens [1, 2], highlighting an ongoing need for therapeutic advancement. However, the expansion of global clinical research has been paralleled by an increasingly complex governance framework capturing all facets of the research process, from trial sponsors to contract research organisations (CROs) and clinical sites. Demanding procedural requirements, excessive data collection and a setup process plagued by in-built redundancy render trials time- and resource-intensive for patients and staff. Repeated calls from multinational healthcare and academic groups for reform [3–5] have sparked some change, but major transformation has been stalled by organisational inertia and an environment that routinely justifies superfluous processes as necessary to patient safety and data quality.

The coronavirus disease 2019 (COVID-19) pandemic triggered a brisk contraction of clinical research in the UK and globally [6]. Cancer patients were promptly identified as a vulnerable group [7], necessitating rapid reappraisal of working practices in both routine cancer care and clinical trials [8]. Mitigation strategies in both sectors centred on rationalisation and decentralisation of services [9]. The transformational experience of setting up and conducting cancer trials in the public sector during the pandemic has helped shine a critical light onto accepted practices.

## Barriers to conduct of clinical trials

The European Union Clinical Trials Directive 2001/20/EC (CTD), whose implementation in 2004 was intended to harmonise trials processes across EU member states by stipulating minimum standards for approval, conduct and monitoring, instead delivered layers of misinterpretation and inconsistency within and between jurisdictions [10]. Subsequent reports cite its more tangible effects as substantially increased trial complexity, cost and workload, disproportionately impeding investigator-led research and eroding an independent public sector approach [11–13]. Despite UK government recognition and review of the ‘unnecessarily complex and burdensome’ regulatory trials framework [11], multiple related issues persisted [14].

Approval of a proposed trial can be a convoluted process of submissions to one or more regulatory agencies, Research Ethics Committees (REC), and institutional research and development (R&D) approval boards generating duplication of duties, conflicting feedback, repeated clarification and redrafting. Since the implementation of the EU CTD, the European Organisation for Research and Treatment of Cancer noted a slowing in initiation of its trials by 5 months, mainly through increased workload for RECs [15]. Acknowledging the issue, regulatory agencies have been moving toward a centralised and integrated approach. These advances nonetheless leave room for R&D departments to continue to engage in redundant assessments overlapping with those delegated to the national boards, a major contributor to delay in trial initiation [11].

Trials are beset by protocol-driven administrative demands on both patients and staff, often disproportionate to the risk posed by the investigational medicinal product (IMP). Rigid trial design and protocols obligate extensive paperwork and reappraisal for sensible and potentially foreseeable alterations. Complexity is most immediately apparent to patients in lengthy trial information sheets and consent forms, potentially disadvantaging those from culturally diverse backgrounds [16]. In early phase (EP) trials, novel therapies are tested conservatively and incrementally to establish safe dosing regimens for subsequent, larger studies of efficacy. Risk varies from highly experimental, first-in-class and first-in-human dosing, to expansion cohorts for established doses of a familiar drug. The density of procedures and checks should be balanced proportionately, but more often clinical review and investigations are mandated with unnecessary frequency. Face-to-face patient contact at least once each week is commonly stipulated for EP trials, and protocols have been known to demand ‘full neurological examination’ more than once between each dose of IMP simply to screen for peripheral sensory neuropathy. Trials of advanced therapies tend to mandate inpatient stays protracted beyond clinical necessity, especially for IMP classes with low incidence of cytokine release syndrome where close outpatient monitoring may suffice. All procedures take place at the investigating site irrespective of the number of adequately equipped facilities between it and the patient’s home, obligating frequent, sometimes lengthy travel. This accessibility issue disproportionately affects socioeconomically disadvantaged populations [17] hence cancer trial cohorts are enriched for wealthier, less ethnically diverse patients living closer to major centres [18, 19]. Patients suitable for EP trials are often in the later stages of their cancer journey, where quality time is paramount and prognosis is generally measured in months [20], in which context excessive clinic attendance may discourage enrolment. Vigilant surveillance and its attendant inconvenience may be warranted in first-in-human, dose-finding scenarios, but once a dose has been established or toxicities already well characterised, the maximally conservative approach should be re-evaluated against drawbacks to patient and investigating site. The proliferation of CROs over the last four decades provides an additional labour pool dedicated to coordinating clinical studies, but as businesses, their fundamental interest—generating capital (sometimes on a per-unit basis)—diverges from those of patients and Investigators and provides little impetus to trim unnecessary procedures [21].

Management of trial databases has come to take up an inordinate amount of time and resources. Detailed clinical information, investigation results and adverse events—many of which will be irrelevant to final publication—are manually transcribed, updated, and clarified in the trial’s data entry system [22]. While this has largely transitioned to electronic formats, each sponsor or CRO tends to use unique, unintuitive software requiring separate training and password-protected accounts. Adverse events (AEs), even those that are trivial, longstanding and unrelated, are followed and queried in detail. Much time is spent responding to requests for arbitrary rephrasing and reformatting, minutiae relating to events both recent and remote, and re-evaluation of causation and grading. Abnormal laboratory parameters are often included as AEs with scope for variable interpretation between investigators across serial tests, generating large volumes of data with a high capacity for inconsistency and subsequent generation of queries. Importantly, a real toxicity signal risks being diluted in this ‘noise’. While careful collection and monitoring of trial data enhances quality and integrity, trial protocols could be much more explicit about which key data points are to be entered into the trial database [23], in turn allowing more proportionate source data verification (SDV). Incremental gains in clinically meaningful information with complete SDV compared to a reduced SDV strategy or centralised statistical monitoring are minimal, in contrast to the extra workload and cost involved; data monitoring consumes up to 25% of the sponsor’s trial budget [23, 24]. Although regulatory authorities have advocated a rationalised, risk-proportionate approach to data monitoring for years [25, 26], the trials community seems to have been reluctant to adopt proportionate SDV as standard.

## Effect of COVID-19 on cancer care and trials, and mitigation strategies

Given the association of cancer with ageing, comorbidities and immunosuppressive treatment, it is unsurprising that oncology patients are overrepresented in morbidity and mortality statistics for COVID-19 [27, 28]. Diversion of healthcare resources to the front-line pandemic response and high rates of staff absence due to isolation diminished the capacity of most cancer centres in 2020 [29]. In this high-risk, resource-constrained environment, non-COVID-related healthcare activities were drastically cut back. UK Cancer screening programmes were suspended, curative-intent surgical procedures were delayed, and the number of patients commencing treatment for screen-detected cancers diminished by 42% [30, 31]. Trials requiring multiple visits were especially susceptible to disruption given a limited pool of trained staff, some of whom were redeployed to newly prioritised COVID-19 trials. Lockdowns interrupted the supply chain for IMPs and laboratory kits as well as site access for patients, trial monitors and regulatory inspectors. Study recruitment was slowed or suspended and new trials launched at only 40% of expected rates in the UK [32] and globally [33].

While the pandemic exposed the vulnerability of the cancer care and research sectors, it also highlighted their adaptability. The cancer community quickly adopted infection risk-mitigation strategies, with rapidly disseminated consensus guidance on balancing therapeutic de-escalation against the potential loss of disease control [34–36]. Algorithms for the use, timing and type of anticancer treatment were re-evaluated, with a higher threshold to proceed where therapeutic benefit was modest or uncertain. In the conventionally fastidious trials sector, regulatory authorities acted early to support unprecedented flexibility in protocol deviations intended to mitigate infection risk and, recognising the imminent overstretch of the healthcare workforce, sanctioned appropriately altered monitoring and quality assurance activities [37, 38]. In response to the pressing need to advance research and therapy for COVID-19, the National Institute of Health and Care Research (NIHR) set up a task force to triage and expedite approvals for new studies into the disease, resulting in unprecedented efficiency in trials setup [39]. Oral IMPs could be directly shipped to patients in both standard care and trials. Supportive therapies to prevent cytopaenia and infection, including granulocyte colony stimulating factor and erythropoietin, were encouraged in anticipation of limited hospital bed space and blood donations. Although decentralisation of procedures and patient consultation is typically avoided in interventional clinical research, COVID-19 prompted the acceptance of remote review for cancer trials patients as telemedicine gained traction across the whole oncology community [40]. Conversion to predominantly remote consultations during the pandemic was associated with higher satisfaction than face-to-face review among cancer patients, mainly due to reduced travel time and expense with greater convenience [41]. This judicious approach extended to trial blood tests, imaging, electrocardiogram and vital signs monitoring, which were revised in frequency and could for the first time be performed at facilities closer to patients. Regulatory authorities were supportive, waiving the litany of paperwork that would have hitherto accompanied such strategies. The longer-term consequences of these temporary strategies warrant monitoring if they are to be extended beyond the pandemic.

Restrictions were placed on outpatient escorts and visitors for inpatients at times of high coronavirus prevalence, with attendant implications for communication and psychological support. These safeguards also precluded trial monitoring visits which, together with pressures on site staff capacity, rendered 100% on-site SDV unfeasible in real time. It was replaced instead by rationalised, remote monitoring of essential data. Though trial recruitment was slowed or halted in anticipation of the expected decline in site capacity, mitigation strategies stabilised and even partially recovered numbers in the face of subsequent waves [42, 43], allowing some trial sites to remain operational in heavily pandemic-affected regions. It was feasible even for some experimental and demanding EP trials to be continued in a safe and scientifically rigorous manner using a risk-adapted approach [44, 45].While these adaptive strategies allowed some cancer trials to stay afloat throughout the ordeal, the rationalised approach was most comprehensively applied to new studies conceived in response to the pandemic itself. The large-scale, adaptive, randomised, UK-based RECOVERY trial for inpatient COVID-19 treatment employed drastically rationalised site set-up and training, consent, eligibility assessment, endpoints, data monitoring and follow-up, along with preferential allocation of large amounts of trials infrastructure and expedient approval, and the use of data linkage. The resulting exceptionally rapid delivery of clinically valuable results is testament to the power of unencumbered clinical research. The trial was launched just nine days from conception, involving tens of thousands of patients across the UK during a time of unprecedented stress on the healthcare system. Within three months it provided world-first evidence of a drug improving survival in COVID-19 [46].

## Future directions for clinical trials—learning lessons from the pandemic

The COVID-19 pandemic launched an unplanned testbed for strategies to streamline clinical trials. The resulting simplification of key research components—the approvals process, site set-up, recruitment and consent, delivery of trial procedures and data handling—together with innovative study design, have proven feasible. They could be preserved beyond the pandemic to optimise the time, resource and financial cost of many trials (Table 1).

**Table 1:** Barriers to and consequences of current trial inefficiencies.

Barrier	Consequence	Responsible body	COVID strategy	Ongoing and proposed mitigation strategies
**Approvals**				
Duplication of procedures between authorities and sites	Time and resource expenditure Delayed trial commencement	Individual site R&D; regulatory authority	Many new trial applications halted to focus on COVID trial set-up	Centralised costing process in motion in UK for commercial studies (NCVR) through interactive costing tool
				Combined MHRA/REC review (IRAS), fast-track REC approval in UK
				Common SOP between similar sites
**Informed consent form (ICF)**				Simplify. Regulatory authorities need to be more specific about information required
Complexity	Suboptimal patient understanding of risks/benefits	Sponsor; regulatory authority in some jurisdictions	Remained complex	Modernised format with less reliance on written documents: e.g. videos, apps, given diversity in literacy rates
				Involve patient groups in ICF design
**Trial procedures**				
Excessive	Time and resource expenditure for investigating site and database management	Sponsor; regulatory authority	Reduction in frequency of some procedures Many non-COVID trials halted	Proportionate reduction in procedure frequency
	Excess time spent in hospital for patients who prioritise quality of life			Prioritise lower-risk trials and patients remaining on trial long term
Mandatory centralisation to investigating site	Geographic inequality in trials accessibility.	Sponsor; regulatory authority	Decentralisation of some investigations, telemedicine. Oral IMP couriered	Telemedicine Acceptance of IMP couriered to patient’s home
	Time and resource expenditure for investigating site			
	Excessive travel for patient			
**Trial design**				
Amendments and protocol deviations generating large volume of paperwork/processes	Time and resource expenditure, trial delays	Regulatory authority, sponsor	Streamlined amendment processes	Built-in protocol flexibility, adaptive design
				Streamlining of amendment processes by regulatory authorities; clarification of minor amendment definition
**Data**				
High volume of low-significance data points collected and queried	Time and resource expenditure for investigating site and monitoring processes	Regulatory authority; sponsor	Many pragmatic protocols, but density of data generated largely unchanged	Clarification and acceptance of proportionate requirements for data analysis Eventual convergence to standard online database, linked to hospital electronic results platforms
Multiple database technologies Excess source data verification (SDV)			Proportionate reduction in SDV requirement	Proportionate reduction in SDV requirement, for example 100% SDV only for DLT period in Phase I trial Incorporation of RWE, data linkage, federated AI systems to enhance study power among increasingly rarer, targeted patient subgroups
**Trial visibility inadequate**	Reduced trial options for patients, slow recruitment	Investigating sites; sponsor; health service	Significant resource made available for recruitment campaigns	Updated trial databases, centralised referrals, incorporation of AI
				Promotion of clinical research as integral part of healthcare provision

### Approvals

The sudden redirection of resources to dramatically expedite approvals for COVID-19 studies during the pandemic is unsustainable as a universal, enduring approach, but efforts by regulatory authorities to streamline clinical trials approvals had been in motion before the pandemic, focusing on centralisation and digitisation of regulatory authority and REC applications [47, 48]. In the UK, initiatives to limit duplication of approvals procedures between sites include centralised costing [49] and pre-emptive assurances for pharmacy and radiation [50]. Without widespread acceptance and trust in this process by individual R&D departments, however, there is a risk of these procedures adding to, rather than streamlining, the existing approvals process.

### Trial design

Improvements in the overarching design of cancer trials have the potential to minimise redundancy and benefit participating patients. Multi-arm platform design with a master protocol and common control group offers the opportunity to efficiently test multiple putative therapies in a single trial with standardisation of procedures across multiple cohorts, and a lower chance of being allocated to control or placebo. This was used to great effect in seminal COVID-19 studies [46]. While examples of this are seen in pivotal cancer trials predating the pandemic—the practice-changing STAMPEDE trial in prostate cancer [51, 52] and the early-phase National Lung Matrix and FOCUS4 trials [53, 54], for example—promoting this approach as standard where feasible would optimise chance of active treatment for trial patients and prospectively facilitate foreseeable updates to trial protocols with minimal unnecessary paperwork. Patient and Public Involvement (PPI) advocates should be involved to ensure that the interests of cancer patients are chief considerations in trial design.

### Informed consent

Simplication of the consent form with PPI input should be encouraged by regulatory authorities and enacted by sponsors. Consideration should be given to alternative forms of information sharing to extensive written leaflets, given variable literacy rates and the surge in digital methods of communication.

### Trial procedures

Major reform in trial procedures requires explicit guidance on appropriate requirements from regulatory authorities, and mutual acceptance from sponsors. Decentralisation of procedures via telemedicine, a clinically valid and cost-effective resource, should be increasingly integrated into both trials and standard cancer care as technological literacy becomes more widespread and digital infrastructure at trial sites continues to modernise. Regulatory recognition of the value of telemedicine, historically a barrier to uptake, would be needed to facilitate this [55]. Delegating investigations to accredited sites closer to patients may similarly minimise unnecessary demands on time and energy for a demographic in which these are of particular value. Portable biological monitoring devices may even provide a means for accurate and convenient measurement of physiologic parameters independent of a healthcare facility, with early trials suggesting feasibility and correlation with clinically meaningful endpoints [56]. These measures to decentralise trial delivery can address regional discrepancies between concentrations of specialist services and patients, diminishing barriers to trial enrolment in underserved populations [57]. Studies have demonstrated that telemedicine is associated with high levels of satisfaction among Oncology patients, and suggested that its use would encourage enrolment to otherwise inaccessible trials [58, 59]. Prospective randomised controlled trials comparing the impacts of a hybrid remote-face-to-face approach with the latter alone on meaningful clinical outcomes would be ideal to validate the approach; such studies incorporating remote reporting of symptoms through a dedicated app have seen significant improvements in overall survival for non-small cell lung cancer patients [60]. Greater geographic and demographic inclusivity is likely to become even more pertinent to contemporary oncology studies as cancer populations are divided into increasingly granular, biomarker-defined subsets and broad genomic profiling becomes standard.

### Data

Along with mutual adoption of a rationalised SDV monitoring culture as standard, evolving artificial intelligence-based approaches to harness the collective power of large swathes of fragmented healthcare data could amplify clinical signals and efficiency of analysis. Real-world evidence (RWE) has been gaining traction in an era of increasingly expedited approvals of targeted therapies based on early-phase trials, as a means to supplant small interventional or control groups, elongate safety follow-up and evaluate generalisability. COVID-19 has accelerated awareness and adoption of RWE to inform clinical practice, although the scientific rigour of this strategy must be scrutinised given the potential for heterogeneity across populations and time [61]. Clinical information siloed across separate medical and registry services can be obtained at the individual patient level for observational studies and clinical trials using data linkage, allowing more extensive follow-up with vastly superior cost-effectiveness [62]. Early in the pandemic, the University of Edinburgh launched the EAVE II project, prospectively linking national healthcare records for over 98% of the population to generate large-scale observational datasets in near-real-time, comprehensively addressing several pandemic-related questions with minimal manual input from an overstretched healthcare workforce [63]. Recently, a rare cancers service in Australia established a nationwide online portal with clinical [64] and analytical research [65] arms. Telemedicine and data linkage are combined to allow patients with rare tumours routine access to highly specialised clinical teams across otherwise prohibitive distances, and also facilitating research of these sparsely distributed populations. Data protection and confidentiality are paramount; sharing between health systems is tightly regulated and particularly complex across international borders. Federated data systems, whereby data held in separate nodes is analysed without leaving its origin, may offer a solution that averts issues with both widespread sharing of sensitive data and duplication of substantial loads of digitised information [66]. The UK Health Data Research Innovation Gateway was established in 2020 as a large-scale, unified portal to access de-identified population-level datasets collected from thousands of individual institutions [67], including hubs dedicated to cancer, COVID-19 and other diseases, and employs federated networks to map available data remotely [68]. These programmes are in their infancy but further development in parallel with improved integration of electronic medical records could herald a new era of progress in observational, interventional and translational research.

## Conclusion

While trials conduct has returned to pre-pandemic practice in the immediate aftermath of lockdowns, there is an opportunity to capitalise on adaptive strategies developed during the COVID-19 pandemic to optimise the delivery of cancer care and clinical trials in future. Rationalised and decentralised procedures, including telemedicine, can improve accessibility of trials, fostering inclusivity of underserved groups reflective of real-world populations, and potentiating research into rare cancer subtypes. Optimised data monitoring can significantly reduce trials costs and workload, and expedite the research process without compromising safety or data integrity. Many of the bolder reforms depend upon explicit clarity and reassurance from regulatory bodies regarding acceptable de-escalation in trial procedural and data requirements, and a culture of mutual trust between authorities, investigating sites and sponsors. Cancer inflicts a devastating global healthcare burden in need of innovative, efficient and safe trials capable of yielding meaningful results in a timely fashion. A competing global healthcare emergency may have given us a glimpse of the way forward.

## Data Availability

Data sharing is not applicable to this article as no datasets were generated or analysed.
